# Can IMTA System Improve the Productivity and Quality Traits of Aquatic Organisms Produced at Different Trophic Levels? The Benefits of IMTA—Not Only for the Ecosystem

**DOI:** 10.3390/biology13110946

**Published:** 2024-11-18

**Authors:** Giusy Rusco, Alessandra Roncarati, Michele Di Iorio, Michela Cariglia, Caterina Longo, Nicolaia Iaffaldano

**Affiliations:** 1 Department of Agricultural, Environmental and Food Sciences, University of Molise, Via De Sanctis snc, 86100 Campobasso, Italy; giusy.rusco@unimol.it (G.R.); michele.diiorio@unimol.it (M.D.I.); nicolaia@unimol.it (N.I.); 2 School of Biosciences and Veterinary Medicine, University of Camerino, Viale Circonvallazione 93–95, 62024 Matelica, Italy; 3 Gargano Pesca Società Agricola Consortile Arl-Società Benefit, Via Rucher 5, Interno 1/C, 71043 Manfredonia, Italy; cariglia.michela@gmail.com; 4 Gargano Shell Fish Farm Societa’ Cooperativa Agricola Arl, Pontile Alti Fondali, SC, 71043 Manfredonia, Italy; 5 Department of Bioscience, Biotechnologies and Environment, University of Bari Aldo Moro, Via Orabona 4, 70125 Bari, Italy; caterina.longo@uniba.it

**Keywords:** improve IMTA acceptability, fed species, extractive species, zootechnical performance, animal welfare, flesh nutritional quality, stakeholders, ESG, One Health

## Abstract

IMTA is one of the most innovative and sustainable farming systems, exhibiting the best technique available in rearing aquatic organisms belonging to different positions along the trophic levels. In the literature and in legislation, the environmental benefits of IMTA protocols have been extensively recognized, mainly for its capability to reduce the ecological footprint of intensive aquaculture systems and concretely address the Sustainable Development Goal no. 14 (SDG 14). However, lesser attention is given to the assessments of its role in enhancing the zootechnical performance, animal welfare, and flesh quality of the species involved. To the best of our knowledge, this is the first review that aims to offer a systematic analysis of the existing literature on the main commercial motivations that could draw the attention of stakeholders, including consumers and fish farmers, towards a greater social acceptability and implementation of the IMTA system on a large scale. The findings suggest that, beyond its environmental advantages, IMTA systems can positively influence the productivity, growth, survival, feed efficiency, and animal health and welfare (AH&W), as well as the nutritional quality of the harvested species, thus offering significant economic and market value both in terms of Environmental, Societal and Governance (ESG) parameters and One Health.

## Simple Summary

This review paper aims to summarize, for the first time, the current state of knowledge on the potential value of the Integrated Multi-Trophic Aquaculture (IMTA) system to positively influence the zootechnical performance, animal welfare, and flesh nutritional quality of the co-cultivated species. The crucial role that the characteristic nutritional (i.e., greater availability of food and nutrients) and environmental factors (i.e., better water quality) of the IMTA system play in shaping the physiological responses of aquatic organisms produced at different trophic levels, i.e., growth, survival, stress responses, and welfare, is highlighted. Given the still-limited diffusion of this valuable fish farm system in many European areas, we believe that emphasizing the economic and market value of IMTA products could contribute to improve public perception and interest in the concrete adoption of IMTA on a commercial scale and constitute a basis to encourage further studies in order to explore new IMTA opportunities.

## 1. Introduction

Integrated Multi-Trophic Aquaculture (IMTA) is one of the most innovative and sustainable farming systems for freshwater, brackish, and marine environments, both in land-based and coastal aquaculture systems [1,2]. Unlike monoculture, which involves the rearing of a single species, and polyculture, where species with similar chemical and biological processes are farmed together, IMTA integrates the synergistic culture of aquatic organisms from different trophic levels [2,3]. In this system, waste products such as uneaten feed, feces, and excretory products of fed species (e.g., finfish, shrimp) become the food for the other organisms, such as organic extractive species (e.g., shellfish, herbivorous fish, echinoderm) and inorganic extractive species (e.g., seaweed) [4,5]. IMTA was primarily developed as a mitigation strategy to address the key environmental concerns associated with intensive aquaculture, including sedimentation and the excessive loading of nutrients and chemicals [6].

In this regard, a conspicuous number of articles and reviews have demonstrated the potential IMTA ability to promote the ecological sustainability [7,8,9,10,11,12,13,14,15,16,17,18,19,20] while delivering economic [21] and social benefits [6]. As an example, many bivalves reduce the organic load of the water by feeding on both phytoplankton and particulate organic matter, leading to significantly control eutrophication. This extractive sequestration of nutrients produces water quality and clarity improvements [22,23]. At the same time, large-scale shellfish–macro-algae IMTA plays an important role in the local carbon cycle and contributes to mitigating ocean acidification and hypoxia [24,25]. Despite the multiple positive effects of IMTA on the environment, this system remains underutilized in many European regions, including the Mediterranean, primarily due to various unresolved challenges [10,26]. Among these, a crucial issue concerns its social acceptability by different stakeholders [6,27]. In this regard, several studies agree on the fact that the development of standards for the definition of a certification of products obtained from IMTA system would allow for greater acceptance by consumers and industries [7,10,24,28]. Others studies conducted on the social sustainability of IMTA also stated that consumers would be willing to pay more for IMTA products if labelled as such [6,29,30]. Moreover, another clear consensus by survey participants was that they wanted to see as much information as possible on the labels, including nutritional facts [6]. Overall, these results confirm the increased attention of consumers in animal-welfare-friendly products, with a low environmental impact and associated with properly tracked and certified quality assurance schemes. Scientifically demonstrating that IMTA models can achieve these production goals would help to address stakeholders and fish farmers to move from a monoculture to an approach based on the IMTA system. Nevertheless, these aspects are often overlooked; indeed, until now, few studies were focused on determining the AH&W and nutritional quality traits of fish, bivalves, and the other organisms involved at the different trophic levels. As concerns farmer’s acceptability, despite the fact that the potential ability of IMTA to provide financial benefits for aquaculture producers has already been widely reported [21], many of them are still against its use, since they do not foresee fast economic feedback, mostly concerning the commercialization of new products such as invertebrates and seaweeds [7]. In this regard, a promising new market application, such as the use of seaweed and polychaete biomass as alternative protein and lipid sources in fish and livestock feeds, could further increase the profitability of IMTA system and positively affect farmer’s acceptance [31,32,33]. At the same time, studies that highlight a high productivity, improved zootechnical performance, and faster production cycles, could be of paramount importance to encourage stakeholder investment and promote a wider application of IMTA systems [17,21].

In the light of these considerations, the aim of the present review is to summarize the current state of knowledge on the potential value of IMTA in producing high-quality nutritional biomass and enhancing zootechnical performances according to SDG 14, which focuses on the conservation and sustainable use of the oceans, seas, and marine resources. Moreover, the application of IMTA as potential system for the production of alternative ingredients in the feeds of aquatic species is also explored. By highlighting these aspects, we aim to enhance public perception and interest in the concrete adoption of IMTA on a commercial scale.

## 2. State of the Art of Successful IMTA Practices on the Zootechnical Performance, Nutritional Traits, Animal Welfare, and Sensory Characteristics of Different Aquatic Species

It is generally accepted that to achieve a successful IMTA system it is essential to undertake an interdisciplinary approach. Researchers and stakeholders with different backgrounds, such as biologists, natural and social scientists, aquaculture engineers, economists, and industry investors, have collaborated and will do so again in the future, in order to establish proper IMTA setups to obtain the maximum economic, social, environmental and productive potential from these systems [10,17]. Therefore, the successful results in terms of zootechnical performance and nutritional quality reported in the following sections are the result of experimental IMTA models planned according to an appropriate combination of the co-cultivated species, suitable population sizes, distance within co-cultured species, sites of culture, and farming methods.

Most of the reported studies agree that the better water quality and the greater availability of food and nutrients in IMTA systems were the major reasons behind the improvement of fish performance, the production of final higher quality biomass, and the faster growth of bivalve. The enhanced water quality in IMTA systems is favoured by different mechanisms implemented by the cultivated species themselves. For example, in IMTA systems with filter feeders, bivalves help lower nutrient load by filtering and absorbing particulate waste, as well as any phytoplankton overgrowth that is triggered by the presence of dissolved nutrient waste [34].Through bioturbation, they can release phosphorus, promoting a greater concentration of Cyanobacteria, which release dissolved oxygen into the water above via photosynthesis, improve nitrification (particularly ammonia oxidation), and enhance the system’s ability to remove ammonia nitrogen [35]. In earthen ponds, bivalves control the density of the microalgae and particulate matter, contributing to a higher transparency and light penetration of the water column, which in turn increase photosynthetic activity with significantly higher dissolved oxygen and carbon sequestration [36]. Also, deposit feeders such as sea cucumber, cultivated below finfish and shellfish farms, can recycle nutrients, decrease organic carbon loads, and bioturbate sediments, potentially limiting anaerobic bacterial growth and the formation of anoxic zones; at the same time, the inorganic nutrients (P and N) they excrete enhance the benthic habitat [34,37,38,39]. Seaweeds are also used to maintain water quality as a supplier of oxygen through the process of photosynthesis during the day and to absorb excess inorganic nutrients [40]. Overall, the favourable water quality in these systems plays a crucial role in the growth and physiological functions of different cultured aquatic species, contributes to better animal health, and to a reduction in the energy power uses for water aeration in pond culture, also contributing to greater profitability [36,41] and toward the assessment in terms of ESG for a wider use of IMTA in aquaculture and maritime spatial planning [42]. At the same time, the high nutritional value of biomass production seems mainly due to the ability of particulate organic matter extractive organisms to recover and incorporate nutrients (i.e., fatty acids [FAs]) contained in the uneaten fraction of aquafeeds supplied to farmed species, which would otherwise be wasted. In this context, new predictive models are being developed in order to support the industry to optimize the nutritional value of cultured species and manage the aquatic environmental eutrophication footprint in conditions of IMTA production [43]. These models effectively predict the feed composition effects on growth and ω-3 FA content in cultured spices, as well as the nitrogen and carbon concentrations generated in the farming environment. Therefore, they will allow us to facilitate the development of novel sustainable feeds by the industry and to enhance the management of environmental impacts. In the fish species mostly farmed in the Mediterranean Sea, such as gilthead seabream and European seabass, the use of sustainable feedstuffs from seafood trimming and processing and aquafeed manufacture (extrusion) has already been adopted to improve the feed conversion rate while minimizing the environmental impact [44,45].

In the following, we present currently available evidence on the IMTA potential to improve the quality and growth performance of various kind of organisms involved at different trophic levels. An overview of IMTA studies referenced in the review is reported in Table 1, underlining the IMTA setup and placement and the main results obtained.

### 2.1. Fed Species

Fish and shellfish are considered among the main fed species in the IMTA [24,75,76] and furnish nutrients to other organisms of the system, in addition to contributing to the economy of the setup [77]. The studies conducted so far, mainly on the zootechnical performance of fed species reared in IMTA systems, have focused on few fish species (current valid name: *Mugil cephalus*, *Argyrosomus regius*, *Chanos chanos*, *Diplodus sargus*, *Epinephelus* spp., *Labeo rohita*, *Planiliza parsia*, *Planiliza tade*, *Sparus aurata*) and two shrimp species (current valid name: *Penaeus vannamei and Peneaus monodon*), with scarce information on the nutritional traits. Some of these articles also include other topics generally few covered in IMTA studies, such as the effect of this innovative aquaculture system on animal welfare, digestive microbiome, and sensory characteristics.

Starting from IMTA systems in brackish water, two recent studies investigated the integration of milkfish *C. chanos* and the white-leg shrimp *P. vannamei* as fed species with oyster [41] or seaweed [47]. Both studies demonstrated positive outcomes in terms of growth and survival rate, using both organic extractive species (*Crassostrea cuttackensis*) and inorganic extractive species (*Enteromorpha intestinalis*). In the first study, Naskar et al. [41] showed that the best growth performance of milkfish (24.66 ± 0.31 g) and shrimp (15.20 ± 0.17 g) and the lowest apparent feed conversion ratio (0.92 ± 0.01) were obtained in the IMTA treatment at the highest oyster density tested (T3—1.8 kg m^−3^), compared to lower densities (T1—0.6 kg m^−3^ and T2 1.2 kg m^−3^). Additionally, the proximate composition analysis of *C. chanos*’ whole body showed the highest protein and fat contents in T3 (58.04 ± 0.73% and 18.18 ± 0.25%, respectively), while in *P. vannamei*, significantly high values were recorded for fat (4.95 ± 0.19%) and ash (10.08 ± 0.18%) in T3 and the control. The authors suggest that the probable reason behind these latter results is the better utilization of feed into body nutrients by reared animals in IMTA than in the polyculture. In a subsequent study [47], the same fed species were integrated with the green seaweed *E. intestinalis*. This setup showed a significantly better growth of *C. chanos* (12.52 ± 0.24 g) and *P. vannamei* (14.77 ± 0.35 g) at higher seaweed biomass density (T3—1.5 kg m^−3^ and T2—1 kg m^−3^, respectively), compared to the control (milkfish: 11.01 ± 0.08 g and shrimp: 11.79 ± 0.58 g). Notably, shrimp survival rates were significantly higher at all seaweed biomass densities tested (T1—70.0 ± 2.5%, T2—80.0 ± 2.1%, T3—85.0 ± 1.7%) compared to the control (52.6 ± 4.8%). This improvement was likely due to the reduced cannibalism occurring in the presence of seaweed, covering 10 to 20% of the surface area, thus providing a hiding zone for *P. vannamei* during the daytime. As regards the physiological status, the blood (red blood cells, white blood cells and haemoglobin) and haemolymph (haemocyanin and total haemocyte count) parameters were significantly improved in fish and shrimp, respectively, under IMTA at harvest compared to the control. It is well known that growth of these two categories of aquatic organisms increase when the environment is favourable. The presence of seaweed also provided a reduction in stress parameters (catalase, superoxide dismutase, and cortisol) compared to the polyculture system, as a result of the consumption of seaweed naturally rich in various bioactive molecules, including anxiolytic agents. Another experiment was conducted in brackish-water earthen ponds to investigate the effect of bivalves (*Meretrix casta*) in halophyte-based IMTA (*Avicennia officinalis* [T1] or *Bruguiera gymnorhiza* [T2]) on the growth performance of the mullet *M. cephalus* and the shrimp *P. vannamei*, used as the fed species [46]. Higher final weight, specific growth rate, protein efficiency ratio, and total productivity were found in shrimp and fish in both halophyte treatments than in the control. The survival rate of *P. vannamei* was significantly higher in IMTA systems (T1—91 ± 1.5%; T2—89 ± 1%) compared to the control (86 ± 1%), while the feed conversion ratio was reduced by 14% and 5% in the presence of *A. officinalis* in *P. vannamei* and *M. cephalus*, respectively. In an IMTA setup similar to that of Kumara et al. [46], the feed conversion ratio was reduced until 22% compared to the polyculture system [48]. In this study, three main fed species, the mullets *M. cephalus*, *P. parsia*, and the tiger shrimp *P. monodon,* were integrated with oyster (*Crassostrea cuttackensis*) and seaweed (*Enteromorpha*) as the extractive species. Here, mullets (*M. cephalus* and *P. parsia*) exhibited a greater growth (*p* < 0.05) compared to that of control ponds, as well as tiger shrimp, although their increase was not significant. At the same time, the total production was 19% higher in IMTA (*p* < 0.05) compared to conventional polyculture. In a subsequent study, Biswas et al. [49] compared polyculture systems (controls) with various IMTA models where three different fed species (*M. cephalus*, *P. tade* and *P. monodon*) were combined with the “water spinach”, *Ipomoea aquatica* (T1), and the oyster *C. cuttackensis* (T2), or both these two extractive species (T3). The growth of tiger shrimp, *P. monodon*, was higher (*p* < 0.05) in T3 (22.17 ± 1.29 g), than in T1 (15.15 ± 0.43 g), T2 (15.96 ± 1.25 g) and the control (14.68 ± 1.28 g). The pooled survival of fishes and shrimp was similar among treatments, including control, whereas the total production was significantly higher in T3 (1695.7 kg ha^−1^), followed by that in T1 (1211.9 kg ha^−1^), T2 (894.2 kg ha^−1^) and the control (505.4 kg ha^−1^). As regards the whole body composition, the crude protein, lipid, and organic matter contents of harvested *M. cephalus* were similar among treatments; crude protein, lipid, ash, and nitrogen-free extract contents of harvested *P. tade* were higher in T1 (60.84 ± 0.16%, 17.22 ± 0.15%, 16.08 ± 0.30%, 9.27 ± 0.21%, respectively), followed by T3, T2, and C; whereas in harvested *P. monodon*, only crude protein content was significantly higher in T1 (56.51 ± 0.10%) and T2 (56.27 ± 0.09%) than that in C (53.83 ± 0.35%) and T3 (54.13 ± 0.20%). Similar results were also observed in a IMTA freshwater system where *L. rohita* was co-cultivated with floating weed, *Wolffia globosa* (T1), freshwater mussel, *Lamellidens marginalis* (T2), or both extractive species (T3) [50]. The total biomass (3641.7 ± 1.33 g), survival (95.38 ± 2.35%), and net fish yield (1.85 ± 0.07 g m^−2^ d^−1^) were significantly high in the IMTA system when Rohu was cultivated with both organic and inorganic extractive species (T3), compared to other treatments and control. The percentage of protein (T1—64.14 ± 0.57, T2—62.67 ± 0.88, T3—62.33 ± 1.51) and lipid (T1—22.17 ± 0.25, T2—20.49 ± 0.24, T3—22.61 ± 0.17) of the whole body of fish was higher (*p* < 0.05) in the all IMTA treatments than the control (protein: 58.71 ± 0.31 and lipid: 17.59 ± 0.41). As regards welfare parameters, the immunological response (nitroblue tetrazolium activity) was significantly higher in T1 (0.50 ± 0.02) and T3 (0.46 ± 0.03), while superoxide dismutase was highest in T1 (6.85 ± 0.34 units/dl serum). The catalase activity was lowest in T3 (0.16 ± 0.03 units/dl serum) among all the treatment groups. Authors suggested that the optimum water quality maintained in the IMTA system protected fish from oxidative stress, thereby improving their immunity and survival.

The enhanced water quality resulting from the IMTA system was also the main reason for the improved fish performance and higher biomass production in the study conducted by Cunha et al. [36]. In this work, the best results were obtained when three fish species (*A. regius*, *D. sargus* and *M. cephalus*), oyster (*Crassostrea gigas* triploid), phytoplankton, and macroalgae (mainly *Ulva spp*.), were integrated in earthen ponds altogether. In particular, phytoplankton played a crucial role by not only increasing dissolved oxygen levels and absorbing excess nutrients from animal waste but also serving as a food source for oysters. Oysters in turn had an important role in regulating microalgae and particulate matter densities in ponds, which helped to maintain stable dissolved oxygen levels and increase water transparency. This enhanced transparency, along with nutrient input from oyster excretion, promoted greater phytoplankton growth, with advantages not only for oysters themselves but also for the macroalgae. At the same time, fish benefited from this good ecological balance, recording higher total final harvested biomass (1115 ± 2.4 Kg) and fish density (1.49 ± 0.01) and lower food conversion rate (1.59 ± 0.00) than other IMTA setups tested.

Another interesting study was conducted by Verdian et al. [40], which cultivated white shrimp (*P. vannamei*) in floating net cages (KJA system) at sea as primary commodities integrated with rabbit fish (*Siganus doliatus*) and the rhodophyte seaweed (*Euchema cottonii*). The results indicated that the growth performance in terms of the final weight (9.63 ± 0.03 g), survival rate (71.00 ± 14.53%), specific growth rate (2.72 ± 0.02%), feed conversion ratio (2.84 ± 0.42%), and total production (2.73 ± 0.33 Kg m^−2^) of white shrimp cultivated in IMTA system were improved compared to shrimp cultivated in a monoculture system. The ability of rabbit fish to eat biofouling attached to the nets’ cage system made it cleaner, promoting regular water circulation. This condition prevented the obstruction of cages and the accumulation of feed residue and metabolic waste, positively affecting shrimp growth.

Omont et al. [51] studied the effect of the IMTA system on the digestive microbiome changes in *P. vannamei* and *C. gigas* for the first time. These authors found significant variations in the digestive bacterial communities of both species under co-culture in the IMTA system compared to a monoculture system. The dominant phyla found in shrimp intestine and oyster gut were Proteobacteria, with a relative abundance of 59.2% and 66%, respectively, followed by Bacteroidetes, Actinobacteria, and Firmicutes. Particularly, within Proteobacteria, a significative variation in the abundance of Alphaproteobacteria communities (Sphingomonodales and Rhodobacterales) occurred in the co-cultured oyster gut due to the consumption of shrimp waste. At the same time, the reduction in particulate organic matter in water caused by the co-cultured oysters has been related to the enhancement of shrimp performance. Moreover, the co-culture of *P. vannamei* and *C. gigas* seem to have promoted healthy conditions that reduced the relative abundance of Vibrionaceae in the intestine of both species compared to monoculture treatments.

Zhang et al. [52] explored the application of IMTA to manage nutrient balance, microbial communities, and antibiotic resistance genes (ARGs) in aquaculture systems, focusing on the cultivation of fish, shrimp, and algae. Specifically, IMTA systems were tested using hybrid groupers (*Epinephelus fuscoguttatus* × *E. lanceolatus*), shrimp (*P. vannamei*), and a rhodophyte seaweed (*Gracilaria bailinae*), with different setups. The tested systems included a control with a monoculture of groupers (C), a fish–shrimp IMTA (FS), a fish–algae IMTA (FA), and a fish–shrimp–algae IMTA (FSA). The presence of *G. bailinae* effectively removed inorganic nutrients (nitrogen and phosphorus) accumulated in water, which in turn significantly promoted growth and enhanced non-specific immunity and glycolipid metabolism in the hybrid grouper, boosting overall fish health and resistance to infections. Although the growth performance of hybrid groupers reared in the IMTA systems were all larger than those in the C system, those in the FSA system were significantly higher (final body weight: 82.33 ± 3.57 g; weight gain: 40.89 ± 2.41 g; percent weight gain: 98.22 ± 3.30%; specific growth rate: 2.28 ± 0.05%) than the control (final body weight: 66.45 ± 2.08 g; weight gain: 25.02 ± 1.12 g; percent weight gain: 60.48 ± 2.36%; specific growth rate: 1.57 ± 0.05%). Compared with monoculture, IMTA significantly changed the structure of the bacterial community, fostering the beneficial effect. Regarding AGRs, even though water quality improved in IMTA systems, they remained persistent, indicating that while IMTA mitigates some issues, it does not fully eliminate ARGs.

Finally, to the best of our knowledge, flesh quality differences in terms of sensory properties and the nutritional value of aquaculture products coming from distinct production systems, including the integrated one, were only compared in a study by Valente et al. [53]. These authors compared the flesh quality traits of gilthead sea bream (*S. aurata*) from extensive, integrated, and semi-intensive systems with ones produced intensively. The integrated system consisted of the association of algae, shellfish, and fish in earthen ponds using the enriched water effluent of a seabass hatchery as a fertilizer to enhance the productivity. Compared to the intensive system, the seabream from IMTA systems developed an intense interorbital yellow band and a shiny orange patch on the gill cover, while the flesh exhibited a darker appearance and more yellowish colour (lower value of L* and higher value of b*, *p* < 0.05), which could be attributed to environmental conditions; the odour of marine/iodine was significantly higher in IMTA, probably due to the ingestion of *Ulvae* that proliferate in those ponds, as suggested by its presence in the sea breams’ stomachs. Body weight was greater (*p* < 0.05) in the IMTA system (464.8 ± 0.1 g) than in the intensive (396.6 ± 57.8 g) and semi-intensive (334.0 ± 50.9 g) systems. Regarding nutritional quality, sea bream from the integrated systems displayed significantly higher levels of docosahexaenoic acid (DHA, C22:6ω3 [7.75 ± 0.92%]), docosapentaenoic acid (DPA, C22:5ω3 [4.15 ± 0.92%]), arachidic acid (C20:0 [0.70 ± 0.72%]) and arachidonic acid (C20:4ω6 [1.05 ± 0.64%]), compared to the intensive system (2.85 ± 2.79%, 1.06 ± 1.40%, 0.13 ± 0.30%, 0.08 ± 0.21%, respectively). The highest levels of eicosapentaenoic acid (EPA, C20:5 ω3 [6.75 ± 0.78%]) and the best ω3/ω6 ratio (2.41 ± 0.67) were also observed in the integrated system, although no statistical significance was found over the intensive system (2.80 ± 2.23%, 0.79 ± 0.95%, respectively).

The success of these IMTA models for the production of the main fed species (finfish and shellfish) is the result of the balanced systems which promoted positive effects on the aquatic environments, animal welfare, harvestable yield, and quality traits, contributing to greater profitability for the producers.

### 2.2. Organic Extractive Species

In IMTA systems, the organic extractive species are used to reduce water nutrient loads acting at different trophic levels according to the type of organic matter generated by fish farms. When it comes to larger organic particles, such as uneaten feed and feces, that settles below the cages, they are eaten by deposit feeders, like polychaetes, sea cucumbers, and sea urchins; while the fine suspended particles are filtered out of the water column by filter-feeding animals like mussels, oysters, scallops, and sponges [5,23]. In a comprehensive book concerning the goods and services of bivalve shellfish [78], bivalves are dealt as filtering nutrients from particulate matter, using the nutrients directly emitted from fish farm in negligible quantities. Thus, when suspended and deposit feeders (for example, bivalves and echinoderms) are combined in IMTA models, the waste removal efficiency of the system tends to increase [17]. 

Among organic extractive species, bivalves (*Crassostrea corteziensis*, *Ostrea edulis*, *Pinctada radiata*, *Mytilus galloprovincialis*, *Mytilus edulis*), echinoderms (*Apostichopus californicus*, *Apostichopus japonicus*, *Holothuria poli*, *Holothuria sanctori*, *Holothuria theeli*, *Holothuria tubulosa*, *Paracentrotus lividus*, *Tripneustes depressus*), polychaetes (*Capitella* sp., *Diopatra neapolitana*, *Hediste diversicolor*, *Ophryotrocha craigsmithi*, *Sabella* cf. *pavonina*, *Terebella lapidaria*), and sponges (*Hymeniacidon perlevis*) have been the subjects of studies aimed at investigating the effect of IMTA systems on zootechnical performance. The ability of the IMTA systems to modify the nutritional traits of produced biomass were addressed only in few studies.

Aguado-Gimenez et al. [54] proposed an experimental integrated culture of flat oyster (*O. edulis*) in combination with an intensive production of gilthead seabream (*S. aurata*) and seabass (*Dicentrarchus labrax*) in open-sea conditions in the southwestern Mediterranean. In this study, the greater availability of food potentially accessible for oysters around the fish farm induced an enhanced development when compared with oysters reared away from the farm. The IMTA conditions not only led to a significantly higher flesh weight with regard to the total weight (i.e., a larger condition index—CI), but also to a more pronounced fattening state and improved FA profile than that observed in the control, which could be interesting from a commercial point of view. The oysters cultured in the integrated area showed significantly higher levels of FAs of terrestrial origin, such as oleic (C18:1ω9 [4.85 ± 0.14%]) and linoleic (C18:2ω6 [2.95 ± 0.06%]) acids, compared to the reference (3.74 ± 0.29%, 2.18 ± 0.14%, respectively), due to the supplement of vegetable oils in food pellets. At the same time, the levels of essential FA, such as EPA (C20:5ω3 [13.82 ± 0.05%]) and DHA (C22:6ω3 [21.54 ± 0.39%]), mainly of marine origin (since they are both very abundant in phytoplankton), were only slightly lower in integrated than in control oysters (14.28 ± 0.18%, 22.67 ± 0.15%, respectively). These results suggest that integrated oysters used particulate wastes derived from the fish farm, but at a low level, because they preferred to use natural seston when available.

The ability to accumulate FAs from uneaten artificial fish feed in tissue was also demonstrated for sea cucumber (*H. roweothuria poli*) co-cultured in coastal Mediterranean IMTA sites directly below the commercial fish cages (*S. aurata* and *D. labrax*) at 10 m and 25 m away [55]. Compared to sea cucumbers cultivated at the reference sites (over 800 m from the fish farm), a high abundance of individual terrestrial plant FAs, such as oleic (18:1ω-9), linoleic (18:2ω-6), and eicosenoic (20:1ω-9) acids, was revealed in sea cucumber tissue near fish cages. At the reference sites, instead, sea cucumber tissues were characterized by a higher relative abundance of arachidonic acid (20:4ω-6) and marine ω-3 PUFAs (EPA and DPA).

These studies, as well as revealing an important bio-mitigation effect of IMTA systems on eutrophication concerns due to the reduced digestibility of plant-derived ingredients by fish, highlights the nutritional benefits of IMTA for oyster and sea cucumber production, where the natural marine resources are not able to sustain their survival and growth.

Relevant results in terms of nutritional quality were also observed for several polychaete species produced through IMTA systems. Recently, Jerònimo et al. [56] reported the FA profile of four different polychaete species (*H. diversicolor*, *D. neapolitana*, *S.* cf. *pavonina*, *T. lapidaria*) cultured in sand filters supplied with effluent water of earthen ponds stocked with gilthead seabream (*S. aurata*) over 15 weeks. In particular, from the FA profile comparison of cultured and wild *H. diversicolor*, an overall dissimilarity of ≈24.2% was revealed, with cultured biomass showing a higher EPA (cultured: 4.83 ± 0.99 µg mg^−1^, wild: 3.68 ± 0.13 µg mg^−1^) and DHA (cultured: 0.99 ± 0.30 µg mg^−1^, wild: not detect) content. Moreover, *H. diversicolor* and *T. lapidaria* were the species whose FA profiles displayed the highest similarity to that of aquafeed formula. Also, other previous works on *H. diversicolor* [79,80] recorded ω-3 highly unsaturated FAs (HUFA) as a major FA class in IMTA cultured (≈34% and 37.8% of total FA, respectively) when the aquafeeds being supplied to fish displayed a higher proportion of HUFA (24% and 20–28% of total FAs, respectively). Overall, these findings demonstrated that polychaetes can be successfully employed to recover otherwise wasted nutrients present in the particulate organic matter of aquaculture effluents, as well as presenting an essential FA profile suitable to be used as ingredients for fish feed formulation.

A study to evaluate the oyster *C. corteziensis* growth cultured under IMTA concept, using the farm effluents of shrimp *P. vannamei*, was conducted by Mazón-Suástegui et al. [57]. Three different growing sowing cycles (October, November, and December), three stocking densities (low, medium, and high), and three positions of the Nestier^®^- like tray (top, middle, and bottom), were investigated in the experiment. The best growth performances were obtained from the October grow-out cycle, from oysters at medium stocking density, and at the top position (wet weight: 110.1 ± 3.6 g, shell length: 91.3 ± 1.5 mm, in 9 months), which far exceeded the typical commercial size (>70 g) of *C. corteziensis* cultured under similar conditions (Nestier^®^ trays suspended from long lines). As was mentioned before, in this case, the greater food availability from the shrimp farm, in the form of particulate organic matter and phytoplankton, also helped to explain the higher growth rates obtained for oysters cultivated under IMTA conditions characterized by warm seawater and low salinity.

Lander et al. [58], investigated the effect of two different distances from two separate Atlantic salmon farms (0 m and 200 m) on the growth parameters (shell length, wet meat weight, and condition index) of blue mussels (*M. edulis*) compared to those grown at a reference site outside the aquaculture influence (control). After one year, an overall significant increase in mussels grown directly on the salmon cages (shell length: 57.47 ± 0.41 mm, wet meat weight: 5.76 ± 0.14 g) was observed, compared to mussels 200 m away (shell length: 54.82 ± 0.61 mm, wet meat weight: 4.62 ± 0.14 g) and control (shell length: 49.50 ± 0.90 mm, wet meat weight: 2.76 ± 0.22 g). This result confirmed what was already observed by the same authors in a previous study, where mussels grew 50% faster in weight when grown in proximity to salmon aquaculture sites [81]. Moreover, mussels held in cages continued to grow in the winter months as well, maintaining a greater meat weight and CI than mussels held 200 m away or at a reference site, which on the contrary recorded a decline in shell growth rate and a decrease in meat weight. This means that mussels at the cage sites gained a more continuous food supply that facilitated early winter growth and enabled overall larger sizes. This phenomenon has also been encountered in the studies by Gvozdenovic et al. [59,82], where higher CI values *of M. galloprovincialis* cultivated close to fish cages (10–100 m distance) were found during a cold period. Overall, the improved growth performance of mussels within and relatively near to fish cages (0 m and 1–100 m distance) under direct organic emission, compared to those grown at larger spatial scales (where there is an increase in particulate dilution), has also been observed in several other papers [60,61,83,84]. In particular, Arduini et al. [60] recently investigated the growth performance of *M. galloprovincialis* under an innovative IMTA system tested in the northern Ionian Sea (Central Mediterranean Sea), that combined fish, mussels and a new group of bio-remediators (sponges, polychaetes and macroalgae), not only in relation to the “horizontal distance” from the cage (1 and 300 m distance), but also to the “vertical distance”, namely on the basis of the water depth (1 and 12 m depth). The CI required for marketing (i.e., 0.15) was achieved only in the mussels grown near the fish cages (1 m distance) and at a depth of 12 m (0.15 ± 0.03), recording CI values among the highest measured in the Taranto area in the last twenty years. Mussels grown in these conditions may have benefited to a greater extent from the organic load of fish cages, which tend to settle on the bottom and/or they may have found temporary shelter from potentially harmful “heat waves” which increased the mussel survival rate compared to those grown at the surface (traditional farming method).

Another innovative IMTA setup was proposed by Chatzivasileiou et al. [61], where fish (*S. aurata* and *D. labrax*), bivalves (*M. galloprovincialis* and *P. imbricata radiata*) and sea cucumber (*H. pollii*) were co-cultured in three fish farms in the Aegean Sea with different trophic conditions. The survival, robustness, and growth parameters of the bivalves and sea cucumber recorded in IMTA system were compared to those of their respective wild populations. All the three species showed high survival rates in the IMTA. The values of CI and meat yield (MY) were higher in IMTA Mediterranean mussels (CI: 33–42%, MY: 19–28%) and pearl oysters (CI: 53–56%, MY: 26–33%) compared to those cultivated under natural conditions (mussels: CI 35%, MY 10%; oysters: CI 30–45%, MY 20–31%). With the Mediterranean Sea being mostly oligotrophic, the areas near fish farms may have been an exception for bivalves due to the significant amount of released nutrients from fish cages. Unlike bivalves, sea cucumber did not gain weight under the IMTA regime, with a final weight reduction even being observed (ranged from 8.3 g to 18.3 g) compared to the initial one (ranged from 59.4 g to 70 g), despite the high survival rate (ranged from 40% to 80%). This last result was perhaps related to the fact that sea cucumbers were not placed directly on the sediment, but tied under the fish cages, which may have protected them from hypoxia. However, factors that negatively affected their development are still under question. 

The efficiency of a similar IMTA setup to that of Chatzivasileiou et al. [61] was also tested in concrete tanks located at the seacoast with an open-flow seawater system [34]. The system was composed of the European sea bass *D. labrax* and organic extractive species from two trophic levels: suspension filter feeders (the mussel *M. galloprovincialis* and the oysters *O. edulis* and *C. gigas*) and deposit feeders (the sea cucumber *H. sanctori*). All filter and deposit feeders showed a 100% survival; the total weight and shell lengths of bivalves were increased in comparison to their initial state (*p* > 0.05), while the initial and final CI ratios were similar. Sea cucumber, instead, displayed a significant increase in total weight at the end of the experiment (132.55 ± 2.58 g) compared to the initial weight (116.81 ± 2.87 g). It is likely that the different cultivation system or physicochemical characteristics of the water positively affected the growth of the sea cucumber, compared to what was observed by Chatzivasileiou et al. [61]. Simultaneously, fed fish showed a significantly lower feed conversion ratio (1.47 ± 0.01) and higher feed efficiency (68.10 ± 0.46%) in comparison to the monoculture system (1.66 ± 0.01 and 60.38 ± 0.24%, respectively).

In light of these studies, it emerged that various factors (i.e., water temperature, salinity, etc.) are responsible for the variation in bivalve’s growth performance cultivated under IMTA systems; however, among these, food availability seems to be the most influential variable.

Because of the important ecological role and high market value, sea urchins and sea cucumber receive increased attention for their use in IMTA. Shpigel et al. [62] conducted practical investigations on the long-term performance of *P. lividus* as an algivorous in a semi-commercial land-based fish (*S. aurata*), seaweed (*Ulva lactuca*) and urchin IMTA system. The benefits of this co-culture for sea urchins included reducing the growth period (reaching commercial size in 3 years rather than 4–5 years), improving gonadal growth, reaching high protein levels in the gonad (ranged between 49.5 and 53.8%) and the generation of three reproductive cycles in one year (two in the winter and one in the spring) rather than one or two seasonal peaks. Researchers attributed these good results to the ad libitum provision of highly nutritious seaweed and relatively constant water temperature throughout the year, which contributed to generate optimal conditions for sea urchins’ growth. 

Regarding sea cucumbers, different species were fed several food sources, such as bivalve feces and pseudo-feces, fish bio-deposits, and sea sediment, in open-water and land-based IMTA systems [37,63,64,65,85]. 

Thanks to their high protein to lipid ratio, high levels of beneficial polyunsaturated FAs, essential amino acids, collagens, vitamins and minerals, sea cucumbers are considered a premium seafood [86]. Paltzat et al. [63] showed that California sea cucumbers (*A. californicus*) held in experimental trays below the cultured *C. gigas* successfully utilized oyster’s bio-deposits (feces and pseudofeces). Sea cucumber showed a mean weight increase of 42.9 g in approximately 12 months compared to the initial weight, and no mortalities in any of the trays deployed during the study were recorded. Positive results were also observed for *A. californicus* of a small size cultivated with sablefish (*Anoplopoma fimbria*) at an IMTA site [37]. Sea cucumbers held below the fish pens grew significantly faster than control animals grown ~250 m away from the farm and recorded a high survival rate (mean 99.5%). High survival (96%) and growth rates were also found for the Japanese common sea cucumber (*A. japonicus*) cultured below a red sea bream cage, compared to the control station [64]. Sea cucumber juveniles reached market size over a culture period of a year and a half (range weight: 142–181 g, mean wet weight: 160 g) and incorporated C3 plant material in fish feed through fish feces. Similar results were also recorded for *A. japonicus* bottom-cultured under a fish farm (main cultivated species: crimson snapper *Lutjanus erythropterus*, blue-spotted grouper *Epinephelus fario*, and cobia *Rachycentron canadum*) in southern China [65]. Debris from the fish cages were efficiently used by sea cucumber, which grew much faster than those at the reference site during winter and early spring.

Overall, these results suggest that co-culture systems among sea cucumber and fish or bivalves would both reduce the amount of organic deposition under farms and produce a valuable secondary cash crop (sea cucumber biomass), promoting the diversification of production.

Combinations of different echinoderm species were also tested in new IMTA setups, where sea urchins are used as primary species and sea cucumbers as the extractive species. An example of this is that reported by Sonnenholzner-Varas et al. [66], based on the co-culture of *T. depressus* (sea urchin) and *H. theeli* (sea cucumber), which was tested to evaluate the use of fresh egested feces of sea urchins that were fed with different natural diets of seaweeds (*Padina durvillaei*, *Sargassum ecuador eanum*, *Kappaphycus alvarezii*, or a mix of these three seaweeds) as food for sea cucumber juveniles. Small (S) and large (L) sea urchins fed with seaweed *P. durvillaei* showed a faster growth (S: 3.0 ± 0.05 cm TD, 7.9 ± 0.2 g; L: 2.1 ± 0.1 cm TD, 5.3 ± 0.1 g, respectively) and lower feed conversion ratio compared to all other treatments. Moreover, sea urchins produced higher amount of feces per day when fed with *P. durvillaei* (L: 31.1 ± 2.7 g d^−1^; S: 11.1 ± 1.2 g d^−1^) and the mixture of seaweeds (L: 28.9 ± 1.9 g d^−1^; S: 10.9 ± 1.2 g d^−1^, *p* < 0.05). At the same time, juveniles of sea cucumber assimilated the feces of sea urchins very well from a diet exclusively based on the mix of seaweed, recording the highest growth rates (length: 7.03 ± 0.03 cm, weight: 5.30 ± 0.11 g) and survival. Overall, the mix of three seaweeds as a natural source of food for sea urchin and its feces (as sub-products) for sea cucumber promoted the proper growth of both organisms. 

Another successful co-culture between two of the most valuable Mediterranean echinoderms (*P. lividus* and *H. tubulosa*) was reported by Grosso et al. [67]. As in the previous study, the effects of three different diets on sea urchin (*P. lividus*) gonad enhancement and somatic growth were evaluated, integrating various levels of fish meal in a land-based vegetable diet—D-0 (100% vegetables, 0% fish meal); D-20 (80% vegetables, 20% fish meal); D-40 (60% vegetables, 40% fish meal). Subsequently, the survival and somatic growth of sea cucumber (*H. tubulosa*) regarding sea urchin bio-deposits of the different experimental diets used as a sole food source for sea cucumbers were evaluated. Among these experimental diets, D-20 was consumed more efficiently, with a lower feed conversion ratio (sea urchin: 3.64 ± 1.39; sea cucumber: 0.41 ± 0.04) and sustained high somatic growth rates for both co-cultured species (sea urchin: 0.12 ± 0.02%; sea cucumber: 0.28 ± 0.05%). A high final survival rate was also recorded for both sea urchin and sea cucumber, suggesting a synergic consumption of the food source and no side effects of co-existence in a same rearing environment. According to the obtained results, the suitability both of the rearing system and the procedures followed is also evident in this case. However, in this study, the use of small amount of an animal protein integrator, such as fish meal, was necessary to complete the nutritional profile of whole vegetable diets and sustain the growth of both species in IMTA system. Nevertheless, the use of land-based vegetables as the main ingredient of the diet led to a significant reduction in feed costs.

The integration of sponges in IMTA systems fulfils complementary functions to other filter feeders commonly applied, such as shellfish, aiding in maintaining lower bacterial loads, reducing disease pressure, and enhancing production. Recent evidence on the feeding behaviour of sponges indicates that dissolved organic matter (DOM) represents the main food source, while particulate organic matter (POM) represents only a small portion of their total intake [87]. Since DOM is not a bioavailable food source for most other heterotrophic organisms, sponges serve a unique and valuable extractive component in IMTA. Considering the dynamics of fed aquaculture, where DOM is generated and its production significantly increases with the inclusion of seaweeds as IMTA components [88,89], this release of DOM can promote the bacterial growth, including pathogenic bacteria. Sponges can mitigate this microbial load both by consuming DOM [87] or removing them [90]. In addition, in an IMTA system, sponges not only can remove potentially harmful particles (e.g., bacteria, viruses, fecal pellets) but also enhance their productivity by using DOM. Indeed, sponges are able to convert DOM into POM (detritus) through the “sponge loop” [91], returning valuable resources to the benthic food chain. This detritus serves as a food source for detritivores such as the commercially valuable sea cucumbers [92]. On account of the results obtained in the experiment conducted by Longo et al. [68], where the sponge *Hymeniacidon perlevis* and the mussel *M. galloprovincilais* were co-cultured in an integrated rearing system, the efficiency of the proposed mussel–sponge model has been demonstrated. Indeed, the abundances of all the considered microbial parameters (e.g., culturable bacteria at 37 °C, vibrios, coliforms, enterococci) in seawater from the integrated rearing system were lower than the control area. Moreover, due to the higher bacterial accumulation exerted by the sponge compared to the mussel, its presence reduces the bacterial load in the co-cultured *M. galloprovincialis*. This finding has practical implications for aquaculture, as the integrated rearing of the investigated filter–feeders could reduce the contamination of edible mussels by harmful bacteria. This helps prevent infections in humans and lowers the risk of mussel diseases, which can cause significant financial losses, ultimately enhancing the safety of seafood production and farming.

### 2.3. Inorganic Extractive Species

In the IMTA cultivation system, seaweed serves as the primary producer at the base of the food chain. They play a crucial role by supplying oxygen through photosynthesis during the day, lowering CO_2_ levels, removing ammonia, and absorbing excess nutrients from the aquatic environment [40]. At the same time the seaweeds are converted into potentially valuable biomass and considered as an important secondary cash crop.

*Ulva* and *Gracilaria* are the most widespread genera due to good large-scale seaweed biomass production and significant revenues for the fish aquaculture when adopting the IMTA approach [93]. An example of the high productivity of *Gracilaria chilensis* species cultivated in a long line near salmon farms in an open-water IMTA system was reported by Abreu et al. [69]. High growth performance (average 1.7 kg m^−2^ fresh weight) was recorded up to 1 km from a 1 ha salmon farm with a production capacity of 1500 tonnes, compared to the control (7 km away from the salmon farm) and the traditional bottom culture. The same authors [70] tested the performance of another species of *Gracilaria* (*G. vermiculophylla*) in a land-based aquaculture pilot scale system for sole (*Solea senegalensis*) and turbot (*Scophthalmus rhombus*). The best results were achieved at the lowest stocking density tested of 3 kg m^−2^, where the seaweed system produced 0.7 ± 0.05 kg (dw) m^−2^ month^−1^ of biomass. The present study recorded higher yields than the one reported by Matos et al. [71] in a similar experiment for *Gracilaria bursa pastoris* cultivated at a stocking density of 5 kg m^−2^ (27.0 ± 15.70 g dw m^−2^ day^−1^). Moreover, the *Gracilaria vermiculophylla* biomass reported a high protein mean value of 37.5% (with highest values of 50%). 

The seaweed *G. bursa pastoris*, coupled with *Chaetomorpha linum*, was also co-cultivated for the first time with a new set of filter–feeder bioremediator organisms, such as the polychaete *Sabella spallanzanii* and the sponge *Sarcotragus spinosulus*, in an IMTA system realized at a preindustrial level in the Gulf of Taranto (southern Italy) [74]. This innovative setup showed interesting growth performances (a mean of specific growth rates of 11%, in short time intervals of 11 and 7 days for *C. linum* and *G. bursa pastoris,* respectively)*,* with high survival rates and a significant amount of biomass produced. 

A more complex system involving the integrated culture of three organisms, abalone (*Haliotis discus hannai*) fish (*S. aurata*), and seaweed (*U. lactuca* or *G. conferta*), in a land-based system, was proposed by Neori et al. [72]. The nutrients excreted by the fish supported high yields of *U. lactuca* (78 kg m^−2^ year^−1^), while *Gracilaria* functioned poorly (only 14 kg annually). In turn, *Ulva* supported an abalone growth rate of 0.9% day^−1^ and a length increase of 40–66 μm day^−1^ in juveniles, and 0.34% day^−1^ and 59 μm day^−1^ in young adults. The total abalone yield was 9.4 kg year^−1^.

Another system, consisting of abalone (*H. discus hannai*) and sea urchin (*P. lividus*) tanks, an intensive fishpond (*S. aurata*), and a three-stage *U. lactuca* biofilter, was proposed by Schuenhoff et al. [73]. During the two experimental periods without (from November to January) and with greenhouse cover (from February to March), *U. lactuca* yield totalled 310 kg (fresh weight) and the protein content averaged above 34% of dry weight. The three-stage biofilter design improved the protein level of the seaweed along with maintaining high nutrient uptake efficiency. The authors suggested that the conversion of nutrient pollution into useful, high-quality seaweed biomass can at the same time offset the costs of biofiltration.

## 3. IMTA as Potential System for the Production of Alternative Ingredients in the Feeds of Aquatic Species

In aquaculture, fish feeding represents a range between 30 and 70% of the total operating costs of the aquaculture production system. It is known that fish meal and fish oil, mainly from small pelagic fish, are the main sources of protein and essential FAs of the ω-3 series, respectively, as well as the most expensive ingredients for the formulation of commercial feeds [94]. Moreover, the overfishing of wild fish stocks for the production of fishmeal and fish oil is one of the major issues in aquaculture sustainability [95]. Therefore, in order to sustain the expected global growth of aquaculture, the search for new protein and lipid sources is of utmost importance. In addition, according to the last FAO Sofia report [96], the aquaculture production has surpassed fishing for the first time; therefore, it becomes increasingly necessary to address the global aquaculture system toward more sustainable models, like IMTA, considering the key role of aquatic foods in climate change. In this regard, the reduction or elimination of dependence on fish-to-feed is also included among the advantages of IMTA [9]. Several studies reported that it is a valid approach for the production of alternative protein and lipid sources for the formulation of aquafeeds. In particular, it has been proven that IMTA systems have been successfully employed to improve the nutritional quality of seaweed [31,97,98,99,100] and polychaete biomass [56] compared to those of wild species, making them more suitable to be used as fish-feed ingredients. Specifically, it has been demonstrated that seaweeds co-cultivated under a high nutrient flux, in addition to promoting higher productivity levels, can increase the protein content over 40% [70,73] and present a high-quality protein profile characterized by a complete essential amino acid profile [100]. Likewise, it seems that polychaetes can be successfully employed to recover and retain protein and valuable HUFA (i.e., EPA and DHA) from uneaten fish feeds that would otherwise be lost to the environment, negatively impacting adjacent aquatic ecosystems [56,79,80,101,102,103].

The production of high-nutritional-value biomass to use as ingredient in feeds represents an important economic incentive for promoting a wider and faster implementation of IMTA systems at a commercial scale. Indeed, the integration of SDG 14 with the parameters related to the circular economy by the exploitation of by-products from IMTA allows us to save costs and environmental benefits [104], and encourages the sector toward the adoption of more sustainable practices and assessment aligned with the ESG parameters, both in a horizontal cross-cut and vertically along seafood production. The cost increase and availability of feeds with proper nutritional composition is on the agenda of FAO, and it is necessary to adopt models like IMTA and enhance technology use throughout the process.

The successful results of the partial replacement of fish meals with seaweed meals that have emerged from the literature seem to be dose- and species-dependent. For the Nile tilapia, several studies investigated the effect of increasing levels of IMTA-produced *Ulva* spp. meal (from 5 up to 30%) in diets at different fish life stages and on various parameters [31,32,98,105]. In particular, the results obtained by Marinho et al. [32], Valente et al. [98], and Silva et al. [105] agree on the fact that the inclusion of *Ulva* spp. meal in diets for Nile tilapia seems to be possible up to 10% without compromising growth performance, body composition, flesh organoleptic properties, and protein utilization and retention. The good capacity of Nile tilapia juveniles to digest and absorb *Ulva* nutrients was suggested from the results obtained by Silva et al. [105], where no significant alterations of gut morphology were observed compared to the control diet. In the same way, with an *Ulva* inclusion level of 30%, Pereira et al. [31] reported the values of protein and energy apparent digestibility coefficients (ADC) most similar to the reference diet, as well as those higher than other tested seaweeds. Moreover, *Ulva* spp. enhanced the innate immune response of Nile tilapia by increasing complement activity (ACH50), promoting the health status of the fish [98]. Overall, these finding suggests that *Ulva* meal could be a suitable partial replacement for fish meal in Nile tilapia diets. 

In California Yellowtail (*Seriola lalandi* syn. *S. dorsalis*) juveniles, the effects of different levels (0.5, 1, and 2%) of dietary supplementation with IMTA-cultivated *Ulva fasciata* on growth performance, haematology, and FA profiles were evaluated [106]. According to the obtained results, *U. fasciata* supplementation up to 2% did not affect fish growth and health. Regarding the haematological parameters, the percentage of haematocrit increased significantly with the higher inclusion of *U. fasciata* meal (46.83 ± 0.76) compared to the control (39.50 ± 3.77), followed also by an increasing trend for red blood cell count (3.19 ± 0.34 × 10^6^ cel mm^3^) and the mean corpuscular volume (150.59 ± 17.32 fL). These results are most likely be associated with the antioxidant compounds contained in seaweed, such as carotenoids and antimicrobial compound ulvan. Also, the total PUFA ω-3 was significantly higher, with a seaweed inclusion level of 1% and 2% in comparison with 0.5% and control, in particular for the value of DHA in the muscle, which increased by approximately 49%. However, considering that this FA was not detected in the *U. fasciata* meal used in this study, the authors assumed that the antioxidants contained in the seaweed meal (e.g., carotenoids) could have prevented lipid peroxidation, preserving the long-chain PUFAs contained in fish feed. 

Another interesting result was reported by Shpigel et al. [107], which showed that high-protein *Ulva lactuca* produced in an IMTA system was proven to be suitable to replace 100% of fishmeal, adding up to 14.6% seaweed in diets for juvenile of *S. aurata*. This percentage of replacements with alga biomass allowed us to obtain a similar performance in terms of SGR (1.44 ± 0.03%) survival (100%) and FCR (1.7 ± 0.05), to those of fish fed the commercial control feed (1.98 ± 0.02%, 100%, 1.5 ± 0.03, respectively), saving almost 10% on the cost of the feed.

The use of *U. lactuca* harvested from a land-based IMTA system was also tested as a partial replacement (25 and 50%) or a supplement (25%) to a commercial shrimp diet for *P. vannamei* juveniles [99]. It was observed that *U. lactuca* can be substituted for up to 25% of a commercial shrimp feed without significantly compromising production performance in terms of growth (2.7 ± 0.26%), survival (81 ± 13.6%), and FCR (2.06 ± 0.49), compared to the control (3.0 ± 0.2%, 81 ± 11.8%, 2.47 ± 0.84, respectively). Moreover, from the nutritional profile comparison between a pelleted diet and *U. lactuca*, it emerged that seaweed contained high levels of protein and an amino acid profile comparable to commercial shrimp feed. This suggests that freshly harvested *U. lactuca* can partially replace a high-protein pelleted diet for shrimp.

A couple of studies on rainbow trout (*Oncorhynchus mykiss*) instead highlight the preference of this species towards *Gracilaria vermiculophylla* seaweed. According to the results by Pereira et al. [31], rainbow trout seems to better digest *G. vermiculophylla* compared to IMTA-cultivated *Ulva spp.* and *Porphyra dioica*, recording an ADC of protein (89.5 ± 0.5%) and lipid (97.5 ± 0.5%) similar to the reference diet (90.3 ± 0.5% and 97.8 ± 0.2, respectively) and significantly higher than other tested diets. In the same way, Araujo et al. [97] observed that the inclusion of *G. vermiculophylla* meal in diets for rainbow trout was possible up to 5%, without impairing gut morphology (intestine diameter and villi height) and thus growth performance. This diet also enhanced the innate immune response of rainbow trout, inducing the highest peroxidase activity (6.86 ± 0.60 U mL^−1^), lysozyme activities (1077.47 ± 161.74 U mL^−1^) and ACH50 alternative complement pathway activity (55.30 ± 6.36 U mL^−1^), compared to the control (4.05 ± 0.37 U mL^−1^, 667.90 ± 54.85 U mL^−1^, 35.85 ± 4.94 U mL^−1^, respectively).

Recently, an innovative mixture of dietary ingredients, that has seen the addition to fishmeal of 10% of polychaete meal (*S. spallanzanii*) and 5% of algal meal (*C. linum*) cultivated by an IMTA system, was tested for farmed juveniles of seabass *D. labrax* [103]. This new formulation, rich in lipids, especially ω-3 PUFAs, showed a survival rate higher than 80% and did not produce negative effects on the fish growth; in fact, no statistical differences were evidenced in biomass gain and specific growth rate between fishes nourished with the control (2.87 ± 0.15 g, 2.96 ± 0.16%, respectively) and the innovative meal (2.1 ± 0.12 g, 2.4 ± 0.11%, respectively). However, as this is a preliminary study, further investigations are needed in order to test higher percentages of *C. linum* and *S. spallanzanii*, aiming to provide fish with additional beneficial compounds and also partially replacing fishmeal.

The potential inclusion of seaweed biomass harvested from the IMTA system in feed is also being considered for livestock, especially rabbits. In fact, a market for algae in the main rabbit-producing countries used in animal feed as alternative to antibiotics already exists. Therefore, the introduction of marine macroalgae coming from IMTA system could be used as a marketing strategy for consumers who are increasingly concerned about issues of environmental sustainability and who are looking for different, high-quality foods [33].

## 4. Considerations and Future Prospects

A very important point emerging from this review was the identification of IMTA system not only as a valuable approach to environmental bioremediation, but also as a method to improve the growth performances and the quality traits of the products and by-products generated in terms of sustainability toward the climate mitigation. In this regard, it emerged that a large body of research literature has been produced in the last two decades, aiming to scientifically prove the potential of various IMTA setups, especially to increase the productivity, and to a lesser extent, the welfare and quality of cultivated species, while reducing waste loadings and environmental impacts. It is known that animal stress and welfare are crucial concerns for producers, as they directly affect the whole-animal’s performance, like survival, growth, reproductive performance, health, susceptibility to diseases [108], and meat quality [109]. Hence, the low presence of studies that support the improvement of animal welfare and sea food nutritional and sensory quality produced by IMTA systems leads us to suggest the development of further joint applied research and projects between universities and industries, aiming to reinforce the results achieved to date. In this regard, proteomics integrated with another “omics” approach has emerged in aquaculture welfare studies as an extremely valuable tool to try to better understand fish welfare, improve welfare status assessment, optimize the fish’s capacity to cope with unavoidable challenges/stress, and find candidate stress biomarkers [110].

According to what was expressed by the EU fisheries policy on aquaculture since 2002 [111], in order to improve the competitive position of the sector and to promote environmental, economic, and social sustainability, it is necessary to find a system able to increase the production and the diversification of species, as well as the product quality. Although the IMTA concept fully meets these requirements, for its wider application, it is imperative that the results of researchers move forward from pilot-scale to commercial-scale development, also taking into account the biosafety of the species involved, as potential risks of contaminant bioaccumulation by extractive species could emerge [76]. In this regard, to enhance their quality and their safety even more, moving forward from open systems to innovative integrated multi-trophic recirculating aquaculture systems (IMTRAS), composed of IMTA and RAS in pairs, is a good strategy [112]. Moreover, it is pivotal to understand that the move to commercial-scale IMTA systems has to also pass through the social acceptability of different kinds of stakeholders. To this aim, the development of a set of production standards and certifications could both increase the consumer confidence in this system and protect producers’ investments. Indeed, precision aquaculture farming could be a possible solution, especially for offshore and open-water IMTA systems, toward the achievement of standardized production methods through the autonomous and continuous monitoring of environmental (i.e., water temperature and tilt, salinity, dissolved oxygen, blue-green algae, chlorophyll, turbidity) and animal variables (parameters related to the behavioural or physiological state of the fish). In particular, this approach applies various technologies and adaptive tools (i.e., wireless sensors, computer vision, AI, underwater video monitoring, hydroacoustic technology, drone, cloud) to monitor, analyze, interpret, predict, and provide more reliable decision support which allows for more effective, precise, accurate, and repeatable farm management [113]. On the basis of these considerations, future studies could be focused on evaluating the possible combination of these two cutting-edge aquaculture practices, precision farming and IMTA, in order to optimize and standardize the productivity, the overall quality of products, and aquatic environmental sustainability, insofar a life below water is considered linked to climate change mitigation. 

In addition, the studies here reported underline the IMTA application as a protocol multivariable, which allows us to increase the market value of by-products obtained along the seafood supply chain by using them as sustainable ingredients to reduce or eliminate aquaculture’s dependence on fish meal and oil. From an economical point of view, the potential inclusion of seaweeds and polychaetes with high quantitative–qualitative proteins and lipids in the feed industrial processing for aquaculture and land-side species can be a promising parallel activity to increase the IMTA system profitability and encourage the companies and policymakers to implement it.

## 5. Conclusions

IMTA showed the potentiality to increase the efficiency and productivity of monoculture systems, adding the possibility of generating products and by-products with improved nutritional profiles and characterized by a high market value, while reducing environmental impact. This productive approach could indeed contribute to achieve SDG 14 and the blue revolution in terms of ESG application in sustainable aquaculture development, which further allows us to apply the recent, viable EU Nature Restoration Law (17.6.2024) [114] that introduces the obligation to restore marine-degraded ecosystems by cross-cutting technologies and bioremediation regarding edible and inedible species. The potential benefits of the IMTA system captured in this review could constitute a basis to encourage further studies in order to explore new IMTA opportunities to improve the public perception of this system and to better understand the commercial motivation to promote the scaling up of experimental IMTA systems towards commercial operations. In this regard, further investigations could be useful to deepen the knowledge of fish farming managed with cutting-edge technological supports in order to provide standardized methods for a high-quality product and environmental protection from the perspective of “precision aquaculture farming” adoption.

## Figures and Tables

**Table 1:** Overview of the IMTA studies referenced in the review.

Fed Species	Organic Extractive Species	Inorganic Extractive Species	IMTA System Placement	Benefits Drawn from IMTA	References
***M. cephalus*** **(mullet)** ***P. vannamei* (shrimp)**	*M. casta* (backwater hard clam)	*A. officinalis* *B. gymnorhiza* (halophyte)	Pond culture	↑ Growth performance (final weight, specific growth rate, feed conversion ratio), survival rate, protein efficiency ratio, total production ↑ Water quality in IMTA = ↑ growth performances	[46]
***C. chanos*** **(milkfish)** ***P. vannamei*** **(shrimp)**	*C. cuttackensis* (oyster)	*-*	Tank culture	↑ Growth performance (apparent feed conversion ratio), protein, fat and ash contents of whole body composition	[41]
***C. chanos*** **(milkfish)** ***P. vannamei*** **(shrimp)**	*-*	*E. intestinalis* (seaweed)	Tank culture	↑ Growth and survival rate ↑ Physiological status (blood and haemolymph parameters) ↓ Stress parameters (catalase, superoxide dismutase, and cortisol)	[47]
***M. cephalus*** **(mullet)** ***P. parsia*** **(mullet)** ***P. monodon*** **(shrimp)**	*C. cuttackensis* (oyster)	*Enteromorpha* (seaweed)	Pond culture	↑ Growth performance (final average body weight, apparent feed conversion ratio), total production	[48]
***M. cephalus*** **(mullet)** ***P. tade*** **(mullet)** ***P. monodon*** **(shrimp)**	*C. cuttackensis* (oyster)	*I. aquatic* (water spinach)	Pond culture	↑ Growth performance (final average body weight, apparent feed conversion ratio), total production ↑ Whole body composition	[49]
***L. rohita*** **(Rohu fish)**	*L. marginalis* (mussel)	*W. globosa* (duckweed)	Tank culture	↑ Growth performance, feed utilization ↑ Welfare parameters ↓ Oxidative stress ↑ Water quality in IMTA = ↑ immunity and survival, ↓ oxidative stress	[50]
***A. regius*** **(Meagre)** ***D. sargus*** **(sea bream)** ***M. cephalus*** **(mullet)**	*C. gigas* (oyster)	*Ulva spp*. (seaweed)	Earthen pond culture	↑ Growth performance (fish density, food conversion rate), total final harvested biomass	[36]
***P. vannamei* (shrimp)** *S. doliatus* (rabbit fish)	*-*	*E. cottonii* (seaweed)	Sea culture	↑ Growth performance (final weight, specific growth rate, feed conversion ratio), survival rate, total production	[40]
***P. vannamei*** **(shrimp)**	***C. gigas*** **(oyster)**	*-*	Tank culture	↑ Growth performance (final weight) Variations in digestive bacterial communities ↓ Relative abundance of Vibrionaceae	[51]
***E. fuscoguttatus*** ***E. lanceolatus*** **(groupers)**	*P. vannamei* (shrimp)	*G. bailinae* (seaweed)	Pond culture	↑ Growth performance (final body weight, weight gain, percent weight gain, specific growth rate) ↑ Non-specific immunity ↑ Liver glycolipid metabolism ↑ Beneficial bacterial communities	[52]
***S. aurata*** **(sea bream)**	Shellfish	Algae	Earthen pond culture	↑ Growth performance (final weight), lipid content (PUFAs ω3), sensory quality	[53]
*S. aurata* (seabream) *D. labrax* (seabass)	***O. edulis*** **(oyster)**	-	Open-sea culture	↑ Condition index, lipid content	[54]
*S. aurata* (seabream) *D. labrax* (seabass)	***H. roweothuria poli*** **(sea cucumber)**	-	Sea culture	↑ Lipid content (PUFAs ω3)	[55]
*S. aurata* (seabream)	***H. diversicolor*** *D. neapolitana* *S.* cf. *pavonina* *T. lapidaria* **(polychaetes)**	-	Sand filters, earthen ponds culture	↑ Lipid content (PUFAs ω3)	[56]
*P. vannamei* (shrimp)	***C. corteziensis*** **(oyster)**	-	Pond culture, channel	↑ Growth performance (wet weight and shell length) exceeded the typical commercial size	[57]
*S. salar* (atlantic salmon)	***M. edulis*** **(mussel)**	-	Sea culture	↑ Growth performance (final wet meat weight, shell length, condition index)	[58]
*S. aurata* (seabream) *D. labrax* (seabass)	***M. galloprovincialis* (mussel)**	-	Sea culture	↑ Condition index	[59]
*S. aurata* (seabream) *D. labrax* (seabass)	***M. galloprovincialis* (mussel)** *S. spallanzanii* (polychaete) *S. spinosulus* *A. aerophoba* *H. perlevis* (sponges)	*C. linum* *G. bursa pastoris* (seaweeds)	Sea culture	↑ Growth performance (shell length, shell dry weight, flesh dry weight, condition index)	[60]
*S. aurata* (seabream) *D. labrax* (seabass)	***M. galloprovincialis* (mussel)** ***P. radiate*** **(pearl oyster)** ***H. pollii*** **(sea cucumber)**	-	Sea culture	↑ Growth performance, survival rate, condition index, meat yield (mussel and oyster) ↑ Survival, ↓ final weight (sea cucumber)	[61]
***D. labrax*** **(seabass)**	***M. galloprovincialis* (mussel)** ***O. edulis*** ***C. gigas*** **(oysters)** ***H. sanctori*** **(sea cucumber)**	-	Sea culture	↑ Survival rate, total weight, shell lengths (mussel and oysters) ↑ Survival rate, total weight (sea cucumber) ↑ Feed conversion ratio, feed efficiency (fish)	[34]
***S. aurata*** **(seabream)**	***P. lividus*** **(sea urchin)**	***U. lactuca*** **(seaweed)**	Tank, pond culture	↑ Gonad somatic growth rate, gonad somatic index, protein level in the gonad, bright orange gonads (sea urchin) ↑ Growth rate, protein and lipid levels (seaweed) ↑ Growth rate, food conversion ratio (fish)	[62]
**-**	***A. californicus*** **(sea cucumbers)** *C. gigas* (oyster)	**-**	Sea culture	↑ Growth rate, survival rate	[63]
*A. fimbria* (sablefish)	***A. californicus*** **(sea cucumbers)**	**-**	Sea culture	↑ Growth rate, survival rate	[37]
*P. major* (red seabream)	***A. japonicas*** **(sea cucumber)**	**-**	Sea culture	↑ Growth rate, survival rate	[64]
*L. erythopterus* (crimson snapper) *E. fario* (blue-spotted grouper) *R. canadum* (cobia)	***A. japonicas*** **(sea cucumber)**	**-**	Sea culture	↑ Growth rate, survival rate	[65]
-	***H. theeli*** **(sea cucumber)** *T. depressus* (sea urchin)	-	Tank culture	↑ Growth rates (length, weight), survival rate	[66]
-	***H. tubulosa*** **(sea cucumber)** ***P. lividus*** **(sea urchin)**	-	Land-based cuture	↑ Growth rates (somatic growth rate, feed conversion ratio), survival rate	[67]
-	***M. galloprovincialis* (mussel)** *H. perlevis* (sponge)	-	Sea culture	↓ Bacterial load in the co-cultured *M. galloprovincialis*	[68]
Salmon	-	***G. chilensis*** **(seaweed)**	Sea culture	↑ Biomass production	[69]
*S. senegalensis* (sole) *S. rhombus* (turbot)	-	***G. vermiculophylla*** **(seaweed)**	Tank, land-based culture	↑ Biomass production ↑ Quality (protein content)	[70]
*S. rhombus* (turbot) *D. labrax* (seabass)	-	***C. crispus*** ***G. bursa pastoris*** ***P. palmata*** **(seaweeds)**	Tank culture	↑ Biomass yield	[71]
*S. aurata* (seabream)	*H. discus hannai* (abalone)	***U. lactuca*** ***G. conferta*** **(seaweeds)**	Tank, land-based culture	Biomass yield ↑ *U. lactuca*, ↓ *G. conferta*	[72]
*S. aurata* (seabream)	*H. discus hannai* (abalone) *P. lividus* (sea urchin)	***U. lactuca*** **(seaweed)**	Tank, pond culture	↑ Biomass production ↑ Quality (protein content)	[73]
-	*S. spallanzanii* (polychaete) *S. spinosulus* (sponge)	***C. linum*** ***G. bursa pastoris*** **(seaweed)**	Sea culture	↑ Biomass production, survival rates	[74]

The target species of the studies are reported in bold. The direction of the arrows shows increase (↑) or decrease (↓) of the effect derived from IMTA setup.
